# Supplementary material for: Nicotine biosynthesis is completed by cryptic activating glucosylation
Source: Nat Commun. 2026 May 18;17:4221. doi: 10.1038/s41467-026-72705-0 (PMC13184244; doi:10.1038/s41467-026-72705-0)
Supplement: Supplementary file 2 — Description of Additional Supplementary Files [file 41467_2026_72705_MOESM2_ESM.pdf]

Description of Additional Supplementary Files

Supplementary Data 1: Genes related to nicotine biosynthesis. Genes from Nicotiana tabacum identified through literature search that are related to nicotine biosynthesis. Top matches between the Edwards et al 2017 genome (https://doi.org/10.1186/s12864-017-3791- 6), the Sierro et al 2024 genome (https://doi.org/10.1038/s41597-024-02965-2) and UniProt accession performed using blast and sequence homology. The Edwards et al 2017 genome gene accessions were used for genome and expression analysis.

Supplementary Data 2: Top correlating genes with A622 in N. tabacum. Pearson correlation coefficients (PCC), calculated from TPM values across 824 samples, extracted from Plant Gene Expression Omnibus. Top 24 highest correlating genes shown in descending order. Names of nicotine biosynthesis genes are added with the newly identified β-glucosidases in bold. Information about gene identity can be found in Supplementary Data 1.

Supplementary Data 3: Top correlating genes with UGT1 in N. tabacum. Pearson correlation coefficients (PCC), calculated from TPM values across 824 samples, extracted from Plant Gene Expression Omnibus. Top 24 highest correlating genes shown in descending order. Names of nicotine biosynthesis genes are added with the newly identified β-glucosidases in bold. Information about gene identity can be found in Supplementary Data 1.

Supplementary Data 4: Nicotine biosynthesis proteins examined in vitro in this study.

Supplementary Data 5: Statistical analysis of in vitro nicotine synthase reactions. EIC peak areas ($n = 3$) for each chemical was compared across in vitro reactions containing different combinations of enzymes and substrates. ANOVA (degrees of freedom [group] = 9; degrees of freedom [residual] = 20) determining peak area difference across reaction combinations was performed, followed by a Tukey HSD post-hoc test, with peak area grouping assigned letters based on comparisons between all samples where $p<0.05$. All reactions contain nicotinic acid. Bar charts can be found in Figure 3.

Supplementary Data 6: Statistical analysis of isotopologue ratios from d4-nicotinic acid fed in vitro nicotine synthase reactions. Isotopologue ratios (d3/d4) were calculated per chemical and per sample through integration and division of EIC peak areas. Samples with zero peak area for either isotopologue were removed from the analysis. Then isotopologue ratios ($n = 3$) for each chemical were compared across in vitro reactions containing different combinations of enzymes and substrates. Analysis of variance determining ratio difference across reaction combinations was performed, followed by a Tukey HSD post-hoc test, with peak area grouping assigned letters based on comparisons between all samples where $p<0.05$. All reactions contain d4-nicotinic acid (2). Bar charts can be found in Figure 5B and S8.

Supplementary Data 7: Data collection and refinement statistics for crystal structure datasets. Numbers in brackets refer to data for highest resolution shells.

Supplementary Data 8: N. tabacum genes transiently expressed in N. benthamiana. Coding sequences of the genes of interest were obtained from the N. tabacum v1.0 Edwards 2017 genome (scaffold) by considering available RNAseq data in the genome browser on the website of the Sol genomics Network (https://solgenomics.net/organism/Nicotiana_tabacum/genome).

Supplementary Data 9: Top correlating genes with A622 in N. benthamiana. Pearson correlation coefficients (PCC), calculated from TPM values across 476 samples, extracted from Plant Gene Expression Omnibus. Highest correlating genes shown in descending order. Gene annotations performed using top blast hits using queries from N. tabacum.

Supplementary Data 10: Statistical analysis of in planta step-wise reconstitution. For each isotopologue (D0, D3, D4) a one-way ANOVA was conducted to test for differences between gene combinations (groups = 7, n = 8, degrees of freedom [group] = 6; degrees of freedom [residual] = 49). The combination with only GFP was set as reference. Post-hoc comparisons were carried out using Dunnett's test (multcomp package, two-tailed, degrees of freedom = 49) to compare each construct directly against the reference group.

Supplementary Data 11: Statistical analysis of in planta drop-out experiment. For each isotopologue (D0, D3, D4) a one-way ANOVA was conducted to test for differences between constructs (groups = 7, n = 8, degrees of freedom [group] = 6; degrees of freedom [residual] = 49). The combination containing all genes was set as reference. Post-hoc comparisons were carried out using Dunnett's test (multcomp package, two-tailed, degrees of freedom = 49) to compare each construct directly against the reference group.
