# Supplementary figures and images for: Nicotine biosynthesis is completed by cryptic activating glucosylation
Source: Nat Commun. 2026 May 18;17:4221. doi: 10.1038/s41467-026-72705-0 (PMC13184244; doi:10.1038/s41467-026-72705-0)

NaGT

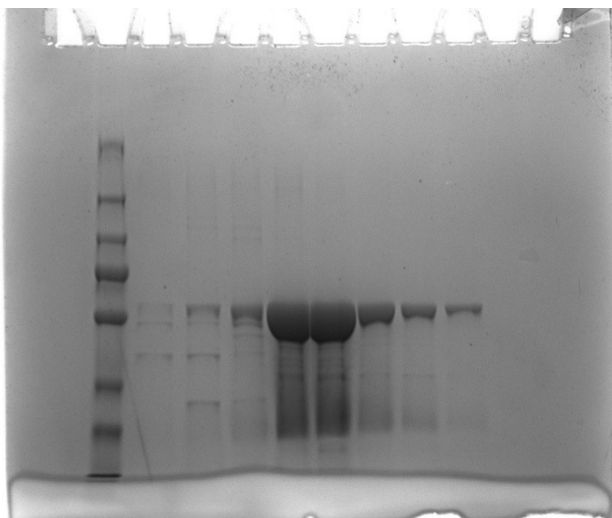

NaGR

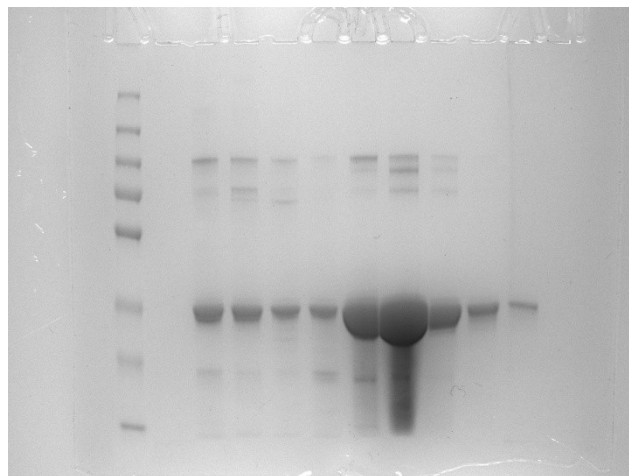

NicGS

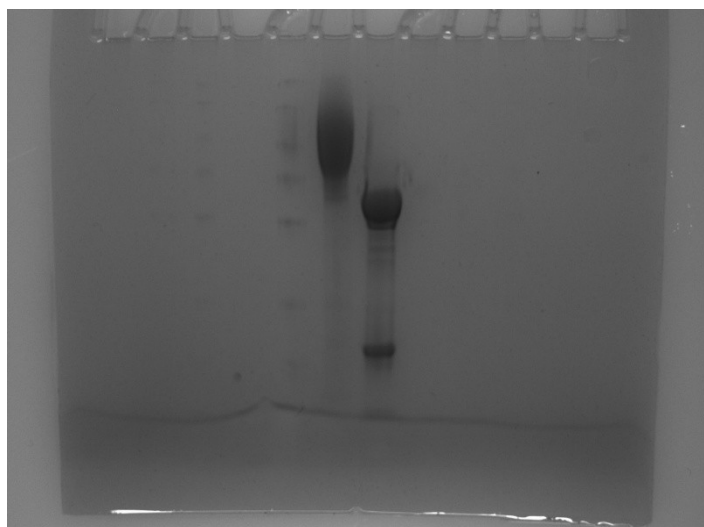

NicGH

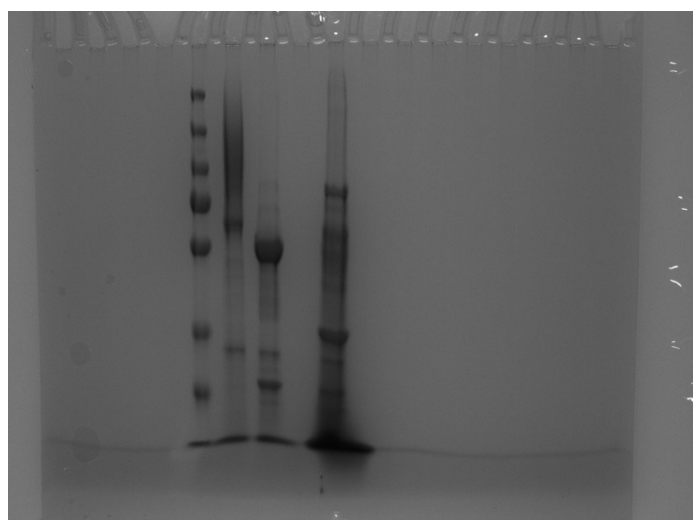

Supplement: Supplementary file 16 — Source Data [file 41467_2026_72705_MOESM16_ESM.zip › Source data_17-04-26/Source Data Fig. S2.pdf]
